# The work environment disability-adjusted life year for use with life cycle assessment: a methodological approach

**DOI:** 10.1186/1476-069X-12-21

**Published:** 2013-03-06

**Authors:** Kelly A Scanlon, George M Gray, Royce A Francis, Shannon M Lloyd, Peter LaPuma

**Affiliations:** 1 Department of Environmental and Occupational Health, School of Public Health and Health Services, The George Washington University, Washington, DC, USA; 2 Department of Engineering Management and Systems Engineering, School of Engineering and Applied Science, The George Washington University, Washington, DC, USA; 3 Concurrent Technologies Corporation, Johnstown, PA, USA

**Keywords:** Disability-adjusted life year (DALY), Life cycle assessment (LCA), Occupational health

## Abstract

**Background:**

Life cycle assessment (LCA) is a systems-based method used to determine potential impacts to the environment associated with a product throughout its life cycle. Conclusions from LCA studies can be applied to support decisions regarding product design or public policy, therefore, all relevant inputs (e.g., raw materials, energy) and outputs (e.g., emissions, waste) to the product system should be evaluated to estimate impacts. Currently, work-related impacts are not routinely considered in LCA. The objectives of this paper are: 1) introduce the work environment disability-adjusted life year (WE-DALY), one portion of a characterization factor used to express the magnitude of impacts to human health attributable to work-related exposures to workplace hazards; 2) outline the methods for calculating the WE-DALY; 3) demonstrate the calculation; and 4) highlight strengths and weaknesses of the methodological approach.

**Methods:**

The concept of the WE-DALY and the methodological approach to its calculation is grounded in the World Health Organization’s disability-adjusted life year (DALY). Like the DALY, the WE-DALY equation considers the years of life lost due to premature mortality and the years of life lived with disability outcomes to estimate the total number of years of healthy life lost in a population. The equation requires input in the form of the number of fatal and nonfatal injuries and illnesses that occur in the industries relevant to the product system evaluated in the LCA study, the age of the worker at the time of the fatal or nonfatal injury or illness, the severity of the injury or illness, and the duration of time lived with the outcomes of the injury or illness.

**Results:**

The methodological approach for the WE-DALY requires data from various sources, multi-step instructions to determine each variable used in the WE-DALY equation, and assumptions based on professional opinion.

**Conclusions:**

Results support the use of the WE-DALY in a characterization factor in LCA. Integrating occupational health into LCA studies will provide opportunities to prevent shifting of impacts between the work environment and the environment external to the workplace and co-optimize human health, to include worker health, and environmental health.

## Background

Life cycle assessment (LCA) is an internationally-recognized systems-based method used to evaluate potential impacts to human health and the environment resulting from extractions or emissions into the environment across a product’s life cycle to include resource extraction, manufacturing, distribution, use, reuse and recycle, and disposal. LCAs are used to make comparative assertions regarding the attributes of one product versus a similar, competing product, to determine the life cycle stage(s) contributing to human health and environmental impacts, or to determine the magnitude of impacts to various areas of concern (e.g., humans, environment, or natural resources). Conclusions and recommendations resulting from LCA can be applied to product design and development, pollution prevention strategies, public policy-making, and marketing (e.g., eco-labeling to support product comparisons). These applications are intended to produce a product with less environmental burden.

Because LCA results are used to support decisions, all relevant inputs and outputs to the product system along the life cycle should be evaluated. To ensure accurate identification of relevant inputs and outputs, life cycle-based studies must be able to evaluate impacts to the environmental, societal, and economic dimensions of products. For example, LCA can be used in conjunction with life cycle cost analyses to evaluate environmental impacts and associated costs. Ongoing efforts to integrate additional dimensions in life cycle-based assessments are important to ensure decisions improve the sustainability of products and lessen potential impacts to human health and the environment [1].

The work environment is potentially as important as the external environment in the assessment of impacts on health and well-being [2] and the health of the workforce is a relevant indicator of sustainability [3]. Efforts to eliminate hazardous materials from the environment through emissions controls can lead to increased exposures to the workers inside the facility generating the emissions [4-6]. For example, regulatory limits on particulate matter, metals, and polyaromatic hydrocarbons emitted from waste-to-energy facilities (also known as incinerators) reduce the overall risk of community health impacts and environmental health impacts [7]. However, the maintenance required to ensure proper operation of the controls increases the likelihood that workers contact these materials during routine maintenance tasks [7,8]. Several workplace exposure assessments have indicated how control technologies designed to reduce environmental emissions worsen the health and safety of workers [5,9]. In addition, efforts to eliminate or reduce hazardous chemicals through substitution of an alternative chemical may introduce new, unknown chemical hazards or new work processes that result in impacts to workers [4,5,10].

Despite this knowledge, occupational health aspects and impacts are not often considered in LCA studies. LCA was not designed or intended to assess occupational health; however, the LCA method is robust and can be expanded to include a variety of impact categories including impacts to human health from the work environment. Several efforts to integrate work environment and occupational health in life cycle-based assessments and integrated product policies have been attempted. Topics addressed include occupational and environmental health impacts of various industries [11,12] and specific product systems including polystyrene products [2] and drilling fluids in offshore crane lifts [13]. The authors of these methods based the occupational health impact assessments on available occupational health and safety statistics for specific industry sectors in specific counties. They used these data to estimate impacts to worker health, measured in disability-adjusted life years (DALYs) or lost workdays, in the industries represented. Methodological limitations highlighted by the authors included the lack of complete, reliable, and consistent occupational health statistics to inform their models. Despite data limitations and uncertainties, the authors concluded that occupational health impacts from the work environment are, in some industry sectors, of the same magnitude or greater than potential environmental impacts assessed for a product system [12,13] or estimates of potential occupational health impacts were large enough to be a consideration in the assessment of total impacts [2,11,13].

The methodological approach presented in this paper builds upon previous work concerning the integration of occupational health impacts in LCA. The objectives of this paper are: 1) introduce the work environment disability-adjusted life year (WE-DALY), a parameter used to express the magnitude of impacts to occupational health; 2) outline the methods for calculating the WE-DALY; 3) demonstrate the calculation of the WE-DALY; and 4) highlight strengths and weaknesses of the approach.

It is expected that the WE-DALY can have a significant role in advancing methods to integrate occupational health impacts in LCA. The WE-DALY, as a ratio to the physical amount produced by the industries represented in a product’s life cycle, can be used to characterize impacts to human health from work environment hazards in those industries. Decision-makers (e.g., regulators or process engineers) who receive results from a life cycle model that includes work environment will be better equipped to evaluate comparative assertions and tradeoffs and promote risk reduction strategies for both the worker and the environment.

### Introducing the Work Environment Disability-Adjusted Life Year (WE-DALY)

In accordance with the International Organization for Standardization (ISO) 14040 and 14044 standards, the LCA method comprises:

• goal and scope definition;

• compilation and quantification of an inventory of inputs and outputs of extractions (e.g., minerals and metals) and emissions (e.g., releases to air, water, or soil);

• classification of input and output flows to impact categories (e.g., stratospheric ozone depletion potential or human toxicity potential), category indicators, and characterization models;

• characterization to evaluate the magnitude of the potential impacts; and

• interpretation of results in relation to the defined goal and scope to draw conclusions and make recommendations [14,15].

LCA applies a structured aggregation procedure to classify the inventory of input and output flows and characterize the impacts to show their magnitude in comparison with other impact categories. Converting life cycle inventory results to impact categories requires a characterization model and a characterization factor, a multiplier which is applied to transform the life cycle inventory results to a common unit for an impact category [14,15]. The WE-DALY is intended to be used in a new work environment characterization factor. It is a single index of impacts to human health that can be used in LCA in characterization of aggregate worker health impact effects.

The concept of the WE-DALY and its calculation is grounded in the World Health Organization’s (WHO) DALY, a measure of the burden of disease that represents a reduction in human function or well-being. The DALY is a measure of the gap between current status and an ideal, optimal health situation where the population lives to an advanced age free of disease and disability [16]. In addition to its use as an indicator of disease burden, the DALY can be used to characterize impacts to human health in LCA. Several life cycle impact assessment methods use the DALY to indicate impacts to human health in LCA studies. In this role, the DALY is used to characterize aggregated impact effects to human health from environmental stressors.

The DALY is an ideal health index to express the magnitude of impacts to human health in LCA because it aggregates different health states over time and space [17]. The life cycle of a product occurs over time and space; it is spatially or geographically indistinct, therefore any changes in health associated with environmental stressors emitted during the life cycle should be similarly spatially or geographically indistinct. The DALY considers the years of life lost (YLL) due to incident cases of premature mortality (YLL) and the years lived with disability (YLD) due to incident cases of disease or injury that impact an individual’s performance.

To calculate the DALY:

(1) DALY=YLL+YLD

For each fatality, the number of YLL is defined as the difference between the actual age at death and the expectation of life at that age in a low-mortality population. For YLD, the years of healthy life lost is obtained by multiplying the expected duration of the condition by a severity weight for the condition. One DALY is considered the equivalent of one lost year of healthy life. The development of the WE-DALY as an impact indicator for human health impacts from occupational aspects is a natural progression and moves LCA toward being a holistic assessment tool.

## Methods

To calculate the WE-DALY, industry-specific work-related fatal and nonfatal injury and illness data are used. Across a product’s life cycle, workers are employed to mine, transport, manufacture, recycle, or dispose of raw materials, the product itself, or its associated waste. In the U.S., the type of work that workers are employed to do, the establishment for which they work, or the product that they produce is identified by the U.S. Census Bureau North American Industrial Classification System (NAICS) code. Industries are identified by a six-digit code. For example, motor vehicle tire manufacturing is classified in the 2007 version NAICS code 326199.

The U.S. Department of Labor, Bureau of Labor Statistics (BLS) collates data from U.S. industries regarding the annual rate and number of work-related fatal and nonfatal injuries and illnesses and organizes these data in accordance with the NAICS. The fatal and nonfatal injuries and illnesses experienced by workers are reported from workplace exposures to chemical, biological, or physical hazards associated with the nature of the work in each specific NAICS.

For each industry or NAICS (n), the number of years of life lost (YLL_n_) is represented by premature mortality in the worker population and for each nonfatal injury or illness the number of years of life lived with disability (YLD_n_) is represented by the severity of the work-related injury or illness and its duration. To calculate the WE-DALY per industry or NAICS:

(2) $$\mathrm{WE}\_{\mathrm{DALY}}_{n}{=\mathrm{YLL}}_{n}{+\mathrm{YLD}}_{n}$$

The remainder of this section provides the methodological approach used to determine the WE-DALY and the data needed for its calculation.

### Years of Life Lost (YLL)

For each industry represented in the product life cycle, the YLL_n_ is based on the number of fatalities reported in a given year and the age at which the fatalities occur. Table 1 lists the data source, data output, and description of the variables.

**Table 1:** Years of life lost data inputs and outputs

**Data source**	**Data output**	**Description**
BLS worker fatality data per industry or NAICS	N_a,s_	Number of fatal workplace injuries per age strata (a) and sex (s)
National Vital Statistics Reports life table data	L_a,s_	Average number of years of life remaining at age of death per age strata (a) and sex (s)

The YLL is calculated as follows.

(3) $${\mathrm{YLL}}_{n}=\sum_{a=1}^{9}\sum_{s=1}^{2}\left(N_{a,s}\;x\;L_{a,s}\right)$$

where N is equal to the number of fatal injuries per age strata (a) and sex (s); L is the average number of years of life remaining at age of death per age strata (a) and sex (s); and, n is the industry or NAICS.

#### Number of fatal workplace injuries

Data for fatal workplace injuries for each six-digit NAICS are available from publicly-available archives and can be obtained online through the Occupational Injuries/Illness and Fatal Injuries Profiles system sponsored by the BLS. Fatality data come from the Census of Fatal Occupational Injury (CFOI) program, a cooperative task between State and Federal governments. States obtain data on fatal work injuries from death certificates marked injury at work, workers’ compensation reports, news media, and U.S. Department of Labor Occupational Safety and Health Administration (OSHA) assessment reports to identify work-related fatal injuries. The fatality is included in the national database only if the State and BLS agree that there is sufficient information on the source document to determine that the case is work-related [18]. CFOI fatality counts exclude illness-related deaths unless precipitated by an injury [18].

For some NAICS, work-related fatal injury counts are unspecified and represented by a dash symbol. These null fatality data do not differentiate between circumstances in which the data from the State did not meet BLS publication criteria or situations where there were no fatalities reported. To avoid losing the NAICS from the assessment because of null data, the number of fatal workplace injuries can be estimated using data available for similar NAICS. For example, to estimate the number of fatalities in a six-digit NAICS, the fatality data in the “sister” six-digit NAICS and the “mother” five-digit NAICS are used. NAICS 33391 comprises NAICS 333911, 333912, and 333913. If data are provided for 333911, 333912, and 33391, then the number of fatalities in 333913 is estimated by subtracting the total number of fatalities in the six-digit NAICS from the total number of fatalities in the five-digit NAICS. In this case, subtraction is used to estimate the count. However, if data are provided for NAICS 33391 only, the number of fatalities in NAICS 333911, 333912, and 333913 are estimated by dividing the total number of fatalities in NAICS 33391 by three. In this example, the estimation process distributes the number of fatalities assuming equal distribution among the NAICS.

The BLS fatality data are categorized into nine age strata: Under 16, 16–17, 18–19, 20–24, 25–34, 35–44, 45–54, 55–64, 65 and over. BLS fatality data per age stratum for each NAICS are used to determine the fatalities for each age stratum. The percentage of fatalities for each age stratum occurring in each sex (N_a,s_), is determined either from the percentage of fatalities occurring in each sex as reported by the BLS or by applying a general percentage based on historic CFOI data trends. The former approach considers each NAICS individually and applies the percentage to each NAICS included in the LCA. The latter approach is based on occupational health and safety data which indicate approximately 93% of workplace fatalities each year occur in the male work population [19-23]. The approach selected depends on the topic of the evaluation (i.e., the product or service) and the assessment’s goal and scope. If the scope includes a broad swath of U.S. industries, then the latter approach is recommended.

#### Years of life remaining

The YLL_n_ calculation considers the number of fatalities occurring in each NAICS with the age at which the death occurred. It is assumed that if the worker did not sustain the work-related injury resulting in premature mortality, then they would have lived for an average lifespan of someone the same age. An estimate of the number of years of life remaining can be made based on data provided in life tables (e.g., U.S. Life Tables published by the National Center for Health Statistics, National Vital Statistics Reports). U.S. life tables are available for specific populations, race, and sex.

The BLS reports fatal workplace injuries per age stratum, not by each individual’s actual age. Therefore, the number of years of life remaining must be estimated for the age stratum. To estimate the average number of years of life remaining for each BLS age stratum, a life table that best represents demographics of the population of concern is used. In the case of the example presented in Table 2, a life table for U.S. males would be used. The life table will provide a value for the expectation of life at each age starting in the first year of life.

**Table 2:** **Example of BLS age stratum and life table overlay **[24]

**BLS age stratum 25 to 34**	**Expected number of years remaining, U.S. Males**
25–26	51.5
26–27	50.6
27–28	49.7
28–29	48.8
29–30	47.8
30–31	46.9
31–32	46.0
32–33	45.0
33–34	44.1
34–35	43.2
Average Number of Years Remaining	47.4

The WE-DALY approach then matches the nine BLS age strata to the selected life table(s) to obtain an average life expectancy for each BLS age stratum. Table 2 provides an example matching the BLS age stratum for age 25 to 34 with life table data for U.S. males [24]. In this example, a male U.S. worker in this age stratum has approximately 47.4 years of life remaining; if a worker in this age stratum died in a work-related accident, the estimated YLL_n_ would be 47.4 years.

The overlay process, as shown in Table 2, is repeated for the remaining BLS age strata. For the age stratum “65 years and over” a life expectancy of 75 years was determined to be the last year for which workplace-related fatality data would apply. This assumption is based on the life expectancy of U.S. males in 2006 [24]. The same method can be used to estimate average number of years remaining for females for each BLS age stratum.

### Years of Life Lived with Disability(YLD)

For each industry or NAICS (n) represented in the product life cycle, the YLD_n_ is based on the number of nonfatal injuries and illnesses reported in a given year, the severity of the injury or illness, and the duration of time that the worker lived with the injury or illness. Table 3 lists the data source, data output, and description of the variables included in the determination of YLD_n_.

**Table 3:** Years of life lived with disability data inputs and outputs

**Data source**	**Data output**	**Description**
BLS worker nonfatal injury, illness data for each NAICS	I_c,a,s_	Number of nonfatal workplace injuries and illnesses for each type of injury or illness (c) per age strata (a) and sex (s)
BLS nature codes from the Occupational Injury and Illness Classification System (OIICS) Manual; WHO severity scores for diseases and conditions	W_c,a_	Severity of each type of nonfatal injury or illness (c) per age strata (a)
For life-long injuries and illnesses: life table data from National Vital Statistics Reports	D_c,a,s_	Duration of life lived with life-long nonfatal injury or illness (c) per age strata (a) and sex (s)
For short-term injuries or illnesses: days away from work with or without job transfer or restriction as provided by BLS	D_c_	Duration of life lived with short-term nonfatal injury or illness (c)

The YLD is calculated using Equations 4a, b, c, depending on the duration of the injury or illness, and Equation 5.

For injuries and illnesses with life-long (LL) duration.

(4a) $${\mathrm{YLD}}_{n,\mathrm{LL}}=\sum_{c=1}^{x}\sum_{a=1}^{5}\sum_{s=1}^{2}\left(I_{c,a,s}\;x\;W_{c,a}\;x\;D_{c,a,s}\right)$$

For injuries and illnesses with short-term (ST) duration.

(4b) $${\mathrm{YLD}}_{n,\mathrm{ST}}=\sum_{c=1}^{x}\sum_{a=1}^{5}\sum_{s=1}^{2}\left(I_{c,a,s}\;x\;W_{c,a}\;x\;D_{c}\right)$$

For injuries with both LL and ST duration.

(4c) $$\begin{array}{c} {\mathrm{YLD}}_{n,\mathrm{LL}+\mathrm{ST}}=\left({\mathrm{YLD}}_{n,\mathrm{LL}}\;x\;\left(\%\;\mathrm{LL}\right)\right)\\ \;+\left({\mathrm{YLD}}_{n,\mathrm{ST}}\;x\;\left(\%\;\mathrm{ST}\right)\right) \end{array}$$

Use Equation 5 to calculate the total YLD_n_.

(5) $${\mathrm{YLD}}_{n}{=\mathrm{YLD}}_{n,\mathrm{LL}}{+\mathrm{YLD}}_{n,\mathrm{ST}}{+\mathrm{YLD}}_{n,\mathrm{LL}+\mathrm{ST}}$$

where I is the number of nonfatal injuries and illnesses for each BLS nature code (c) reported for the nonfatal injuries and illnesses occurring in each NAICS for each age strata (a) and sex (s); W is the severity weight assigned to the nature code for each BLS nature code (c) reported for the nonfatal injuries and illnesses occurring in each NAICS and for each age strata (a); D is the duration of the illness; LL represents injuries and illnesses with life-long duration; ST represents injuries and illnesses with short-term duration; n is the industry or NAICS; and, x represents the total number of nonfatal injuries and illnesses incurred by the study population.

#### Number of nonfatal injuries and illnesses

Data for nonfatal work-related injuries and illnesses involving days away from work (with and without job transfer or restriction) are available from the BLS. Nonfatal data are reported to the BLS from the Survey of Occupational Injuries and Illnesses (SOII), a survey that includes injury and illness data provided by most, but not all, U.S. establishments each year [18]. The SOII measures the number of new work-related injury and illness cases which are recognized, diagnosed, and reported by employers.

The BLS Occupational Injury and Illness Classification System (OIICS) Manual is used as a guide by U.S. government employees and industry representatives responsible for reporting and coding case characteristics of injuries, illnesses, and fatalities in the SOII, as well as the CFOI. The data reported include information from injury and illness logs in accordance with OSHA recordkeeping requirements.

Examples of case characteristics include nature of injury or illness, part of body affected, source of injury or illness, and event or exposure [25]. In determining the YLD_n_, the case characteristic nature of injury or illness is used to determine the severity of the injury or illness.

Null nonfatal injury and illness data for a specific NAICS in a given year does not differentiate between circumstances in which the data did not meet BLS publication criteria and situations where there were no nonfatal injuries or illnesses. To avoid removing these NAICS from the assessment because of null or missing data, the number of nonfatal injuries and illnesses can be estimated using data available for similar NAICS. The estimation process is previously described in the section addressing the number of fatal workplace injuries.

To determine the number of nonfatal injuries and illnesses for each nature code and for each sex and age stratum requires additional manipulations of the BLS nonfatal injury and illness data, specifically, the creation of a weighted multiplier. A weighted multiplier distributes the number of incident cases of nonfatal injuries and illnesses as identified by a nature code across age strata for each sex. The apportioning of nonfatal injuries and illnesses is necessary because a severity weight will be assigned to each nature code based on the severity of the injury or illness, the age at which the injury or illness occurred, and the duration of time lived with the injury or illness.

To calculate the weighted multiplier requires four steps. First, BLS age- and sex-stratified data are obtained from tables of nonfatal injuries and illnesses requiring days away from work. BLS uses nine age stratifications for nonfatal injuries and illnesses per sex: Under 14, 14–15, 16–19, 20–24, 25–34, 35–44, 45–54, 55–64, and 65 and over. Table 4 provides an example of age- and sex-stratified nonfatal injury and illness data for U.S. private industry in 2006 [26].

**Table 4:** **Number of nonfatal injuries and illnesses **[26]

**BLS age strata**	**Females**	**Males**
< 14	0	0
14 to 15	30	140
16 to 19	13270	26040
20 to 24	39070	92890
25 to 34	80640	189250
35 to 44	100750	200060
45 to 54	101640	163970
55 to 64	53210	80200
65 >	10490	14660

Second, the age- and sex-stratified data are parsed to distribute the number of nonfatal injuries and illnesses occurring each year of life with the assumption that there is even distribution of nonfatal injuries and illnesses each year. For example, in 2006, BLS reported that females age 25 through 34 experienced a total of 80,640 nonfatal injuries and illnesses [26]; it is assumed that 8,064 nonfatal injuries and illnesses occur for each year for each of the 10 years of this age stratum.

Table 5 lists assumed age- and sex-stratified nonfatal injury and illness data. The data used in this example are for U.S. private industry in 2006; this example uses the same data presented in Table 4. In this example, age-stratified data were parsed with the assumption that females and males will not work beyond their expected lifetime (i.e., 80.2 years for females and 75.1 years for males) [24].

**Table 5:** Example of parsed BLS age- and sex-stratified nonfatal injury and illness data

**BLS age strata**	**Number of years in each age stratum**	**Distribution of nonfatal injuries and illnesses, Females**	**Distribution of nonfatal injuries and illnesses, Males**
< 14	14	0	0
14 to 15	2	15	70
16 to 19	4	3318	26040
20 to 24	5	7814	6510
25 to 34	10	8064	18925
35 to 44	10	10075	20006
45 to 54	10	10164	16397
55 to 64	10	5321	8020
65 to 80 (females)	16 (females)	656	1333
65 to 75 (males)	11 (males)		

The third step required to calculate the weighted multiplier is the distribution of the BLS age- and sex-stratified data to match with WHO age strata. This step is required because the severity weights that will be assigned to the nature codes are based on the WHO’s Global Burden of Disease (GBD) severity scores for diseases and conditions [16]. The WHO severity scores are age-stratified to represent burden of disease at various points in the human life cycle. There are five WHO age strata: 0–4, 5–14, 15–44, 45–59, and 60 years and over. Table 6 lists parsed stratified nonfatal injury and illness data per WHO age strata. The data used in this example are for U.S. private industry in 2006; this example uses the same data presented in Table 5. In this example, the age-stratified data are distributed using the same lifetime assumptions applied earlier.

**Table 6:** Example of WHO age- and sex-stratified nonfatal injury and illness data

**WHO age strata**	**Females**	**Males**
< 4	0	0
5 to 14	15	70
15 to 44	233745	508310
45 to 59	215835	204070
60 to 80 (females)	56765	54760
60 to 75 (males)		

Finally, the WHO age-stratified and sex-stratified data are used to determine the weighted multiplier. The weighted multiplier is equal to the percent of injuries and illnesses occurring per age strata and sex. For example, there were 1,166,310 total nonfatal injuries and illnesses reported 2006. Of these, 233,745 (20%) were experienced by females age 15 through 44 and 508,310 (44%) were experienced by males age 15 through 44. The weighted multiplier for females age 15 to 44 in 2006 is 0.20 and the weighted multiplier for males age 15 to 44 in 2006 is 0.44. Table 7 lists the age- and sex-stratified weighted multipliers for nonfatal injury and illness data for 2006; this example uses the same data presented in Table 6.

**Table 7:** Weighted multipliers for nonfatal injuries and illnesses

**WHO age strata**	**Weighted multiplier, Females**	**Weighted multiplier, Males**
< 4	0.00	0.00
5 to 14	0.00	0.00
15 to 44	0.20	0.44
45 to 59	0.11	0.17
60 to 80 (females)	0.03	0.05
60 to 75 (males)		

The weighted multiplier is then applied to the nonfatal injury and illness data to distribute the number of incident cases for each nature code and for each age stratum and sex. To estimate the distribution for each nature code, the total number incident cases for each nature code is multiplied by the weighted multiplier for each age stratum and sex. Table 8 illustrates the distribution of incident cases of nonspecified injuries and disorders (Nature Code 097) for NAICS 113310 for 2006. Data for nonfatal workplace injuries are available from archives available online through the Occupational Injuries/Illness and Fatal Injuries Profiles system sponsored by the BLS. The weighted multipliers presented in Table 7 are used in this example.

**Table 8:** Example of application of weighted multipliers to determine distribution of nonfatal injuries and illnesses

**NAICS**	**BLS Nature Code**	**Number of cases**	**WHO age strata**	Weighted multiplier, females*	**Estimated cases, females**	Weighted multiplier, males*	**Estimated cases, males**
113310: Logging	097: Nonspecified injuries and disorders	100	0 to 4	0.00	0	0.00	0
			5 to 14	0.00	0	0.00	0
			15 to 44	0.20	20	0.44	44
			45 to 59	0.11	11	0.17	18
			60 and over	0.03	3	0.05	5

* Total for females and males may not add to 100% due to rounding.

#### Severity of nonfatal injuries and illnesses

The BLS publishes information on the nature of the injury and illness as determined by the employers who are tasked to complete the SOII. The nature of injury or illness identifies the principal physical characteristic(s) of the work-related injury or illness; it is categorized into one of eight Divisions and assigned a nature of injury or illness code [25]. To determine YLD, the severity of each illness and injury must be estimated by assigning it a weight. This approach uses the severity scores assigned by the WHO’s GBD studies for diseases and conditions, referred to as sequelae. The BLS nature codes are matched with the GBD sequelae. Then, the severity scores for the sequelae are applied to the nature codes to produce the severity weight.

The severity score/nature code matching process incorporates approximately 200 sequelae (e.g., diseases and conditions, cancers, and injuries) used in the GBD estimates [16]. Sequelae are the effects or conditions resulting from prior disease or injury. The GBD sequelae include: communicable diseases and conditions (e.g., HIV/AIDS); noncommunicable diseases (e.g., hypertensive heart disease and asthma); pre-terminal and terminal cancers; and injuries (e.g., fractures and amputations). The sequelae used in the matching process include those diseases, conditions, and injuries that could be experienced as work-related nonfatal injuries or illnesses. Examples of sequelae that may be judged as work-related include: pathogenic bloodborne diseases; noncommunicable diseases such as hearing loss; and injuries such as fractures and dislocations.

The BLS nature codes are categorized into Divisions: traumatic injuries and disorders; diseases; multiple diseases and disorders; and cases where there is insufficient information to select any nature code [25]. Within each Division, nature codes are assigned to a Major Group and identified using a two-digit code. The branching continues to Group Titles which are assigned three-digit codes and Specific Conditions which are assigned four-digit codes. For example, within Traumatic Injuries and Disorders (Code 0) there is a Major Group identified as Open Wounds (Code 03), a Group Title identified as Amputations (Code 031), and a Specific Condition identified as Amputations, fingertip (Code 0311).

Case definitions of sequelae provided in GBD studies [27] and descriptions and examples of nonfatal injuries and illnesses provided in the OIICS Manual [25] are used to match the sequelae and nature codes. Nature codes at the Group Title level (i.e., three-digit code) and Major Group (i.e., two-digit code) provide the least ambiguous of the various levels of nature codes.

Table 9 lists examples of matches between nature codes and sequelae. The notes listed in Table 9 highlight the challenges faced when matching nature codes with sequelae. In several instances, there is good alignment (e.g., one-to-one match or several sequelae could be grouped to represent one nature code). However, in the majority of matches, an assumption is needed in order to justify the match. These assumptions required judgment about the severity of the nature code and how well it aligned with the sequela. A BLS nature code may represent a class of disorders but the sequela matched to the nature code is narrower in scope. For example, symptoms involving the respiratory system and chest may represent a broad category of illnesses; however, in the methodological approach introduced here, it was matched with the GBD Sequelae for ischemic heart disease and angina pectoris, two defined diagnoses with narrower interpretations.

**Table 9:** **Examples of matches between nature codes and sequelae **[25,27]

**BLS Nature Codes**	**GBD sequelae**	**Notes**
012 Fractures	Fractures: face bones; vertebral column; rib or sternum; pelvis; clavicle, scapula, or humerus; ulna or radius; hand bones; femur; patella, tibia, or fibula; ankle; foot bones; skull	Match.
043 Bruises - Contusions	Open wound	Sequela for bruises and contusions is not available. Assumption: sequela for open wound represents nature code.
097 Nonspecified Injuries and Disorders	Low back pain: episode of limiting low back pain and episode of intervertebral disc displacement of herniation	Nonspecified injuries include low back pain. Assumption: sequelae represent nature code.
180 Disorders of the Skin and Subcutaneous Tissue, Unspecified	Skin diseases, cases	The use of the term “unspecified” in the nature code means that there was no mention of a specific injury to the worker’s body. Assumption: sequela represents nature code
416 Symptoms Involving Respiratory System and Chest	Ischemic heart disease: angina pectoris	Nature code describes symptoms to include chest pain. Assumption: sequela represents nature code.
521 Anxiety, Stress, Neurotic Disorders	Panic disorder, cases; Post-traumatic stress disorder, cases; Obsessive-compulsive disorder, cases	Match.

Then, the severity scores for the sequelae are assigned to the matched nature codes. Age-specific severity scores for sequelae as published in the 1996 GBD reports are used [16]. The severity scores are on a scale of zero (0) through one (1), with 0 representing the least severe sequelae and 1 representing the most severe sequelae. Severity scores for each of the five WHO age strata are assigned to each sequela; the severity of the disease or condition may vary depending on age. For example, sprains are assigned a severity score of 0.064 for all age strata, however retinopathy associated with diabetes mellitus has a higher severity score for younger age strata (e.g., 0.491 for age 5 to 14) and a lower severity score for older age strata (e.g., 0.488 for age 15 to 60 and over) [16]. In addition, the severity scores are classified by treatment form (i.e., untreated or treated).

The severity scores for the sequelae are applied to the nature codes to which they were match to produce a severity weight for each nature code for each WHO age stratum. The YLD_n_ is based on severity scores for treated forms of sequelae. These scores are used because it is assumed that workers who experience a work-related nonfatal injury or illness will receive some type of treatment for the injury or illness.

When the severity score/nature code match is based on a grouping of sequelae, the severity scores for that group are averaged. For example, the severity scores for 12 different types of fractures were averaged to determine the severity weight for Nature Code 012: Fractures. Table 10 lists the severity weights for the matched nature codes presented in Table 9. An additional file lists commonly-occurring BLS nature codes matched to GBD sequelae and the resulting severity weights [see Additional file 1].

**Table 10:** **Examples of severity weights for nonfatal injuries or illnesses per WHO age strata **[16]

**BLS Nature Codes**	**Severity weights**				
	**Age < 4**	**Age 5-14**	**Age 15-44**	**Age 45-59**	**Age 60 >**
012 Fractures	0.311 (LL)	0.311 (LL)	0.311 (LL)	0.311 (LL)	0.338 (LL)
	0.226 (ST)	0.226 (ST)	0.225 (ST)	0.225 (ST)	0.225 (ST)
043 Bruises - Contusions	0.108	0.108	0.108	0.108	0.108
097 Nonspecified Injuries and Disorders	0.061	0.061	0.061	0.061	0.061
180 Disorders of the Skin and Subcutaneous Tissue, Unspecified	0.056	0.056	0.056	0.056	0.056
416 Symptoms Involving Respiratory System and Chest	0.095	0.095	0.095	0.095	0.095
521 Anxiety, Stress, Neurotic Disorders	0.092	0.092	0.093	0.093	0.093

Abbreviations: LL, life-long duration; ST, short-term duration.

#### Duration of time lived with nonfatal injury or illness outcomes

GBD sequelae are categorized as communicable and noncommunicable diseases and conditions and injuries [27]. To determine the duration of time lived with nonfatal injury and illness outcomes, the categories were assigned a duration code: life-long (LL) or short-term (ST). Life-long injuries and illnesses result in permanent disability (e.g., amputations). Short-term injuries and illnesses do not result in permanent disability (e.g., sprains).

For all communicable diseases and conditions, the ST duration code is applied. This duration assignment is based on the assumption that these diseases and conditions do not result in permanent disability. For all noncommunicable diseases and conditions, the LL code is applied. This duration assignment is based on the assumption that these diseases and conditions are chronic and result in life-long disability.

The assignment of duration codes to the injuries category is based on GBD severity scores and durations for injuries in which injuries are assigned as either short-term, life-long, or, in the case of three injury categories, partial short-term and life-long [16]. The three injury categories with partial assignment are: fractured skull, fractured femur, and intracranial injury. The GBD assumed that approximately 15% of fractured skull injuries result in life-long disability and 85% result in short-term disability and, for fractured femur and intracranial injury, 5% of the incident cases result in life-long disability and 95% result in short-term disability [16]. In Table 10, Nature Code 012 (Fracture) has two sets of nature code weights, one for LL and ST. The severity weight for fractures with life-long duration is derived by averaging the severity scores for two different life-long fracture sequelae: fractured skull and fractured femur. The severity weight for fractures with short-term duration is derived by averaging the severity scores for 12 different short-term fracture sequelae.

##### Life-long nonfatal injury or illness outcomes

For injuries and illnesses assigned LL duration codes, it is assumed that workers live and work with disability outcomes for the remainder of their expected lifetimes. To determine the duration of time lived with nonfatal life-long outcomes, the expected number of years of life remaining is estimated using data from life tables and using a method similar to the one applied in the YLL calculation by matching the five WHO age strata to the life table data. The results of the overlay for the WHO age strata are presented in Table 11. U.S. life tables for females and males are used in this example [24]. The values presented in Table 11 will be used in the calculation of YLL_n_ when life-long sequelae are associated with the injury or illness.

**Table 11:** **Life-long duration codes **[24]

**WHO age strata**	**Average number of years lived with life-long outcomes, females**	**Average number of years lived with life-long outcomes, males**
< 4	78.6	73.6
5 to 14	71.3	66.3
15 to 44	51.8	47.4
45 to 59	30.7	27.2
60 to 80 (females)	16.4	15.7
60 to 75 (males)		

##### Short-term nonfatal injury or illness outcomes

For injuries and illnesses assigned ST duration codes, it is assumed that workers who had returned to work no longer experienced the disability from the injury or illness. To determine the duration of time lived with nonfatal short-term outcomes, the median days away from work with or without job restriction or transfer is used. This value is provided by the BLS with the nonfatal injury and illness data. Median days away from work is a measure used to summarize the lengths of absences among cases with days away from work with and without job transfer or restriction; half of the cases reported involved more days away and half of the cases involved fewer days than the specified median value [28].

The values for median days away from work is converted to an annual basis and weighted to account for the assumption that the nonfatal injury or illness is experienced by the worker seven days per week (365.2 days per year) instead of the traditional five work days per week (250 days per year). A multiplier is estimated for this conversion.

(6) $$\frac{365.2\;\mathrm{days}\;\mathrm{per}\;\mathrm{year}}{250\;\mathrm{work}\;\mathrm{days}\;\mathrm{per}\;\mathrm{year}}=1.46\;\mathrm{days}$$

For every one work day in a year, there are 1.46 calendar days. For example, a worker who experiences a nonfatal injury or illness requiring 20 days away from work actually experienced the disability associated with the injury or illness as many as 29.4 calendar days (20 days × 1.46 conversion factor).

Then, the number of calendar days is converted to an annual basis by dividing by 365.24. In this example, 29.4 divided by 365.2 is 0.08; 29.4 days away from work is equivalent to 0.08 years away from work due to disability resulting from the injury or illness. Unlike the other variables in the WE-DALY equation, there are no sex- or age-stratified durations for short-term disabilities.

##### Partial life-long and short-term nonfatal injury or illness outcomes

For injuries and illnesses with partial assignment to both LL and ST duration codes, the methods previously described for calculating D_c,a,s_ and D_c_ are applied. To calculate the YLD, percentages are used to weight the YLD results to allocate the years of life lived with life-long outcomes or short-term outcomes. To determine the applicable percentages requires averaging all LL durations and all ST durations for sequelae that are matched to a nature code.

For example, 12 different types of bone fracture sequelae were grouped together to represent Nature Code 012 (Fractures). Ten of the 12 fracture sequelae have ST durations and two of the 12 have both ST and LL durations (e.g., skull and femur fractures). According to the GBD studies, approximately 5% of femur fractures result in life-long disability and 95% result in short-term disability; approximately 15% of skull fractures result in life-long disability and 85% result in short-term disability [16]. Therefore, on average, 98% of the time the incident case coded as Nature Code 012 results in a short-term outcome and 2% of the time it results in a life-long outcome. Table 12 illustrates this example.

**Table 12:** **Example of partial duration percentages **[16]

**Nature Code 012, fractures**	**Percent of fractures resulting in life-long duration outcomes**	**Percent of fractures resulting in short-term duration outcomes**
Radius or ulna	0	100
Hand bones	0	100
Patella, tibia, or fibula	0	100
Ankle	0	100
Foot bones	0	100
Face bones	0	100
Vertebral column	0	100
Rib or sternum	0	100
Pelvis	0	100
Clavicle, scapula, or humerus	0	100
Femur	5	95
Skull	15	85
Average	2	98

The process for determining percentages is repeated for all nature codes comprising multiple injury durations, mixtures of communicable (ST duration) and noncommunicable (LL duration) diseases and disorders, or mixtures of communicable diseases (ST duration) and injuries (both LL and ST durations). An additional file lists the duration categories and codes assigned to the nature codes including the percentages applied to the partial LL and ST nonfatal injury and illness outcomes [see Additional file 1].

## Results

The YLL_n_ and YLD_n_ are influenced by the industries represented by the product life cycle. The number of fatal and nonfatal injuries and illnesses will vary as will the demographic statistics of the population of workers in these industries.

### Years of Life Lost (YLL)

The following steps were used to determine the YLL_n_:

1. Consult BLS data to determine the number of fatal work-related injuries per BLS age stratum and sex (N_a,s_) for each industry.

2. Use life table data to determine the average number of years of life remaining in each of the BLS age stratum (L_a,s_).

3. For each industry or NAICS (n), multiply the number of fatal work-related injuries per BLS age stratum and sex (N_a,s_) by the average number of years of life remaining per age stratum and sex (L_a,s_) to determine the total years of life lost.

Table 13 presents the results of the YLL_n_ calculation for one industry selected for this example. The data used in this example represent fatalities occurring in 2006 as reported in the CFOI and made available by the BLS for NAICS 113310 (Logging) [20]. In the NAICS selected for this example, 100% of the fatalities occurred in the male population. The average number of years remaining for each age stratum was determining using the matching process, as shown in Table 2, repeated for the remaining age strata.

**Table 13:** **Calculation of years of life lost **[20]

**BLS age strata**	**Number of fatalities (N**_**a,s)**_**for NAICS 113310**	**Average number of years remaining (L**_**a,s**_**)**	**Years of life lost (YLL**_**n**_**) for NAICS 113310**
<16	--*	68.3	--
16 to 17	--	59.4	--
18 to 19	--	57.5	--
20 to 24	7	54.3	380
25 to 34	11	47.4	521
35 to 44	27	38.4	1037
45 to 54	23	29.2	673
55 to 64	17	21.1	359
65 to 75	11	13.6	150
Total	96	N/A	3120

* Dashes indicate no data reported or data that do not meet BLS publication criteria.

Abbreviations: N/A, not applicable.

To calculate the total YLL_n_ for each NAICS, values were added across the age strata. The results in this example indicate there were 96 fatal work-related injuries reported in male U.S. workers in NAICS 113310 in 2006 and 3,120 years of life lost due to these fatalities. If females incurred fatalities, then the N_a,s_ and L_a,s_ for females would be added to the N_a,s_ and L_a,s_ for males to determine the total YLL.

### Years of Life Lived with Disability (YLD)

The following steps were used to determine the YLD_n_:

1. Consult BLS data to determine the number of nonfatal work-related injuries and illnesses per WHO age stratum and sex (I_c,a,s_). Use a weighted multiplier to distribute the number of incident cases for each nature code across WHO age strata and sex. The weighted multiplier is determined as follows:

a. Compile BLS nonfatal injury and illness data for all industries in a given year to determine the total number of nonfatal injuries and illnesses occurring in each of the nine BLS age strata per sex.

b. Distribute the BLS age- and sex-stratified data to determine an assumed number of nonfatal injuries and illnesses occurring each year of life.

c. Redistribute the number of nonfatal injuries and illnesses occurring each year to align with the five WHO age strata.

d. Determine the percentage of nonfatal injuries and illnesses occurring per WHO age strata and sex. The percentage is the weighted multiplier.

2. Use BLS nature codes and WHO GBD severity scores for sequelae to determine the severity weights for nonfatal injuries and illnesses for each age stratum (W_c,a_). To do this, match the nature codes with the sequelae and assign the severity score for the sequelae to the matched nature code.

3. Use WHO GBD sequelae categories to classify nature codes as LL or ST duration. For injuries and illnesses classified as LL duration, use life table data to determine the average number of years of life remaining in each of the five WHO age stratum for each sex (D_c,a,s_). For injuries and illnesses classified as ST duration, use BLS data for median number of days away from work with or without job transfer or restriction to determine the number of years of life lived with nonfatal short-term outcomes (D_c_). Use a multiplier to convert days away from work to years away from work. For injuries classified as both LL and ST duration, calculate D_c,a,s_ and D_c_ and apply the appropriate percentages to weight the contribution.

4. For each industry or NAICS (n), multiply the number of nonfatal work-related injuries for each nature code per WHO age stratum and sex (I_c,a,s_) by the severity weights for each nature code per WHO age stratum (L_a,s_) by the duration of injury or illness for each nature code (D_c,a,s_ or D_c_) to determine the total number of years of life lived with disability outcomes.

Table 14 presents the results of the YLD calculation for an injury with LL duration, specifically Nature Code 097 (nonspecified injuries and disorders). The data used in this example represent nonfatal injuries occurring in 2006 for NAICS 113310 as reported in the SOII and made available online through the Occupational Injuries/Illness and Fatal Injuries Profiles system sponsored by the BLS. The incident cases were distributed by age and sex using the weighted multipliers for nonfatal injury and illness data for 2006 presented in Table 7. The severity weights for Nature Code 097 were previously presented in Table 10 and the LL durations were previously presented in Table 11. The results in this example indicate that, in 2006, there were approximately 248 years of life lived with life-long outcomes due to nonspecified injuries and disorders experienced by workers in NAICS 113310.

**Table 14:** **Calculation of years of life lived with life-long outcomes **[16]

**Sex, WHO age strata**	**Number of cases (I**_**c,a,s**_**) occurring in BLS Nature Code 097, NAICS 113310**	**Severity weights (W**_**c,a**_**) for Nature Code 097**	**Life-long duration code (D**_**c,a,s**_**)**	**Years of life lived with life-long outcomes (YLD**_**n,LL**_**)**
Females, 0 to 4	0	0.061	78.6	0
Females, 5 to 14	0	0.061	71.3	0
Females, 15 to 44	20	0.061	51.8	63
Females, 45 to 59	11	0.061	30.7	21
Females, 60 to 80	3	0.061	16.4	3
Males, 0 to 4	0	0.061	73.6	0
Males, 5 to 14	0	0.061	66.3	0
Males, 15 to 44	44	0.061	47.4	127
Males, 45 to 59	18	0.061	27.2	29
Males, 60 to 75	5	0.061	15.7	5
			Total YLD_n,LL_	248

Table 15 presents the results of the YLD_n_ calculation for an injury with ST duration, specifically Nature Code 043 (Bruises-contusions). The data used in this example represent nonfatal injuries and illnesses occurring in 2006 as reported by the SOII and made available by the BLS for NAICS 113310. The incident cases were distributed by age and sex using the weighted multipliers presented in Table 7. The severity weights for Nature Code 043 were previously presented in Table 10 and the calculation used to determine the short-term duration was presented in the methods discussion. The results in this example indicate that, in 2006, there was less than one year of life lived with short-term outcomes due to bruises and contusions experienced by workers in NAICS 113310.

**Table 15:** **Calculation of years of life lived with short-term outcomes **[16]

**Sex, WHO age strata**	**Number of cases (I**_**c,a,s**_**) occurring in Nature Code 043, NAICS 113310**	**Severity weights (W**_**c,a**_**) for Nature Code 043**	**Short-term duration code (D**_**c**_**)**	**Years of life lived with short-term outcomes (YLD**_**n,ST**_**)**
Females, 0 to 4	0	0.108	0.02	0
Females, 5 to 14	0	0.108	0.02	0
Females, 15 to 44	18	0.108	0.02	< 1
Females, 45 to 59	10	0.108	0.02	< 1
Females, 60 to 80	3	0.108	0.02	< 1
Males, 0 to 4	0	0.108	0.02	0
Males, 5 to 14	0	0.108	0.02	0
Males, 15 to 44	39	0.108	0.02	< 1
Males, 45 to 59	16	0.108	0.02	< 1
Males, 60 to 75	4	0.108	0.02	< 1
			Total YLD_n,ST_	< 1

Table 16 presents the results of the YLD_n_ calculation for an injury with both LL and ST duration, specifically Nature Code 012. The data used in this example represented nonfatal injuries and illnesses occurring in 2006 as reported by the SOII and made available by the BLS for NAICS 113310. The incident cases were distributed by age and sex using the weighted multipliers presented in Table 7. The severity weights for Nature Code 012 were previously presented in Table 10 and the calculations used to determine the percentages of LL and ST durations were presented in Table 12. Approximately 2% of the fractures are estimated to be life-long and 98% are estimated to be short-term. The results in this example indicate that, in 2006, there were approximately 50 years of life lived with outcomes due to fractures experienced by workers in NAICS 113310.

**Table 16:** **Calculation of years of life lived with outcomes of life-long and short-term durations **[16]

**Sex, WHO age strata**	**Total number of cases (I**_**c,a,s**_**) in Nature Code 012, NAICS 113310**	**Number of cases with Life-long duration**	**Severity weights (W**_**c,a**_**) for Nature Code 012, Life-long duration**	**Life-long duration code (D**_**c,a,s**_**)**	**Years of life lived with Life-long outcomes (YLD**_**n,LL**_**), NAICS 113310**	**Number of cases with short-term duration**	**Severity weights (W**_**c,a**_**) for Nature Code 012, short-term duration**	**Short-term duration Code (D**_**c**_**)**	**Years of life lived with short-term outcomes (YLD**_**n,ST**_**), NAICS 113310**
Females 0 to 4	0	0	0.311	78.6	0	0	0.226	0.18	0
Females 5 to 14	0	0	0.311	71.3	0	0	0.226	0.18	0
Females 15 to 44	34	1	0.311	51.8	11	33	0.225	0.18	1
Females 45 to 59	18	< 1	0.311	30.7	3	18	0.225	0.18	< 1
Females 60 to 80	5	< 1	0.338	16.4	1	5	0.225	0.18	< 1
Males 0 to 4	0	0	0.311	73.6	0	0	0.226	0.18	0
Males 5 to 14	0	0	0.311	66.3	0	0	0.226	0.18	0
Males 15 to 44	75	2	0.311	47.4	22	74	0.225	0.18	3
Males 45 to 59	30	1	0.311	27.2	5	29	0.225	0.18	1
Males 60 to 75	8	0	0.338	15.7	1	8	0.225	0.18	< 1
				Total YLD_n,LL_	43			Total YLD_n,ST_	7

Years of life lived with disability for an industry (YLD_n_) is determined by adding together the YLD for each life-long, short-term, and partial life-long/short-term duration (see Equation 5). From the examples provided in Tables 14 through 16, there were approximately 298 years of live lived with disability for workers in NAICS 113310.

### Work Environment Disability-Adjusted Life Year (WE-DALY)

To calculate the WE-DALY for each NAICS, the YLL_n_ and YLD_n_ values are combined (see Equation 2). In the examples presented in this paper, the YLL for NAICS 113310 in 2006 was estimated at 3,120 and the YLD was estimated at 298. Therefore, the WE-DALY is 3,417; there were 3,417 years of healthy life lost due to premature workplace fatalities and nonfatal injuries and illnesses in NAICS 113310 in 2006.

## Discussion

The methodological approach for the WE-DALY presented in this paper requires data from various sources, multi-step instructions to determine each variable used in the WE-DALY equation, and assumptions based on professional judgment. Challenges include data reliability and validity. For example, the WE-DALY is influenced by the number of nonfatal injuries and illnesses occurring in each industry relevant to the product system assessed in the LCA study. The BLS data used to determine the number of nonfatal workplace injuries and illnesses are used based on the assumption that it captures 100% of the actual nonfatal injuries and illnesses that occurred in the U.S. for that industry in a given year. However, several professional, academic, and governmental organizations, including the BLS, have documented that SOII estimates undercount workplace injuries and illnesses [29-35]. An estimate of the undercount of nonfatal injuries and illnesses ranges from 0% to 70% [35].

For some NAICS, work-related fatal injury counts and nonfatal injury and illness counts are unspecified and represented by a dash symbol. These null fatality data do not differentiate between circumstances in which reported data did not meet BLS publication criteria or situations where there were no fatalities of nonfatal injuries and illnesses reported. To avoid losing the NAICS from the assessment because of null data, the number of fatal workplace injuries can be estimated using data available for similar NAICS. The choice to estimate these counts is based on the assumption that the injuries and illnesses occurred in the NAICS for which null data were reported. Also, the estimation process distributes the number of injuries and illnesses equally among the missing “sister” NAICS based on data provided for the “mother” NAICS. Equal distribution may not represent actual cases. The estimation process may be enhanced through occupational health and safety expert elicitation regarding work environment hazards and the incidence of work-related fatal and nonfatal injuries and illnesses.

Cases of nonfatal injuries and illnesses are recordable and reportable in the SOII if the injury or illness resulted in death, days away from work, restricted work, transfer to another job, loss of consciousness, medical treatment beyond first aid, or significant injuries or illnesses diagnosed by a physician or other licensed health-care professional. The SOII is completed by employers; several disincentives that influence the employers’ decisions to record or report work-related injuries and illnesses have been documented. They include the difficulty in determining whether some illnesses are work-related (e.g., those with long latency periods, like cancer) and the lack of understanding of recordkeeping requirements by those who are responsible for recording work-related nonfatal injuries and illnesses [33].

The methodological approach for the WE-DALY can accommodate the potential undercount of nonfatal injuries and illnesses. When determining the number of incident cases, a multiplier can be developed and implemented to reflect what the research believes to be a more realistic representation of actual cases. Then, several YLDs can be calculated to represent a range of valid counts. For example, if it is assumed that the nonfatal injury and illness data undercount the actual number by 50%, then a multiplier can be used to ensure all reported nonfatal injury and illness counts are doubled. The resulting YLD will be larger than if the data are assumed to represent 100% of the actual incident cases; the profile of NAICS included does not change, however the magnitude of impact for each industry will increase.

Nonfatal injury and illness data include the nature of the incident case (e.g., fractures or respiratory system diseases); these data are used to determine the severity weights for nonfatal injuries and illnesses and the duration of life lived with the outcomes of the injury or illness. The severity score/nature code matching process and associated assignment of severity weights does not need to be repeated for each LCA study. However, LCA practitioners may opt to repeat the matching exercise to confirm the judgments made in the approach presented in this paper or to produce their own matches and associated severity weights based on their own assumptions. Likewise, they may opt to review the durations assigned to the nature codes to confirm the assignments already made or produce their own duration assignments.

U.S.-based workplace fatality and nonfatal injury and illness data are used to inform the WE-DALY. However, workplace health and safety statistics from other countries can be used in the equation. Ideally, the occupational health statistics used in the WE-DALY would reflect the product’s life cycle stages, to include a global chain of resource extraction, manufacturers, transporters, users, and recycling and disposal locations. In the absence of global occupational health statistics, U.S.-based statistics can be applied. However, U.S. occupational health statistics will reflect the U.S. economy and the prevalence of U.S. health and safety standards and best practices and, therefore, may not accurately represent the workers in the global chain for that product system.

Another set of assumptions that can affect the approach is the selection and use of discounting and age-weighting – both of which stem from the methods for determining the DALY originally promoted by the WHO in their GBD studies [16]. When estimating DALYs, a discount rate for valuing future benefits can be applied. Discount rates value a year of healthy life saved now more than a year of healthy life saved years from now. Higher discount rates value future health losses less. Also, age-weighting schemes can be applied to the DALY calculation to account for the relative value of a year of healthy life lived at different ages. Age-weighting regards a healthy year of live lived at a young age less than that of someone who is in their mid-20s. The GBD studies completed in 2004, the most recent year for which data are published, used 3% discounting and non-uniform age weighting, a practice that give less preference to years lived at very young and very old ages.

DALYs in LCA differ from their application in the WHO GBD estimates. LCA takes into account emissions of all pollutants with a toxicity potential across the life cycle of the product regardless of where and when they were released to the environment; in LCA, past, present, and future years of life lived are equal. Because LCA does not differentiate the timing of release, exposure, or health effects, typically a discount rate of zero is applied in the calculation of the DALY in LCA [17]. A similar philosophy can be applied to the calculation of the WE-DALY. The methodological approach for the WE-DALY can be extended to include discounting and age-weighting: discounting would be applied during the calculation of the YLL and age-weighting would be applied to both the YLL and YLD calculations. Discounting and age-weighting schemes have not been developed for DALYs used in LCA. However, the approach promoted by the WHO GBD studies may be able to be adopted for use in LCA.

## Conclusions

The WE-DALY is a new index created to express the magnitude of impacts to human health resulting from work-related exposures occurring across the life cycle of a product system. The WE-DALY can be incorporated into a work environment characterization factor, a ratio of the WE-DALY to the physical amount produced by the industries represented in a product’s life cycle. This new characterization factor would be used in LCA to assess impacts to human health from work environment hazards. The approach presented in this paper builds upon existing ideas concerning the integration of occupational health assessment in LCA. Integrating occupational health into LCA studies will provide opportunities to prevent shifting of impacts between the work environment and the environment external to the workplace and co-optimize human health, to include worker health, and environmental health. Methodological limitations discussed include data reliability and validity, nuances of the calculation, generalizability outside the U.S. supply chain of products, and researcher-based assumptions. However, these limitations should not deter occupational health or LCA practitioners. Occupational health statistics for countries represented by global supply chains can be coordinated from national agencies, when available, or estimated using historical data from U.S. and other countries where the collection of data is prevalent and its dissemination well-documented. To account for data limitations, uncertainty analyses and sensitivity analyses can be conducted and documented. For those limitations that cannot be remedied, data sources, assumptions, and limitations should be documented. Future research studies will focus on the application of the WE-DALY as a characterization factor in LCA.

## Abbreviations

BLS: Bureau of Labor Statistics; CFOI: Census of Fatal Occupational Injury; DALY: Disability-adjusted life year; GBD: Global Burden of Disease; ISO: International Organization for Standardization; LCA: Life cycle assessment; LL: life-long duration; NAICS: North American Industrial Classification System; OIICS: Occupational Injury and Illness Classification System; OSHA: Occupational Safety and Health Administration; SOII: Survey of Occupational Injuries and Illnesses; ST: Short-term duration; U.S.: United States of America; WE-DALY: Work environment disability-adjusted life year; WHO: World Health Organization; YLD: Years lived with disability; YLL: Years of life lost.

## Competing interests

The authors declare that there are not conflicts of interest.

## Authors’ contributions

KAS conceived and developed the methodological approach, the name WE-DALY, and drafted the manuscript. GMG, PL, RAF, and SML participated in the drafting and revising of the manuscript. GG critically reviewed the draft. All authors read and approved the version to be published.

## Supplementary Material

Additional file 1 **Severity Weights and Duration Codes.** The additional file lists commonly-occurring BLS nature codes matched to GBD sequelae and the resulting severity weights. Also, the file includes duration categories and codes assigned to the nature codes including the percentages applied to the partial LL and ST nonfatal injury and illness outcomes. Click here for file

## References

[B1] Lloyd S Scanlon K Lengacher D Subic A, Wellnitz J, Leary M, Koopmans L Improving life cycle assessment by considering worker health and comparing alternatives based on relative efficiency Sustainable Automotive Technologies 2012 Proceedings of the 4th International Conference: 21–23 March 2012; Melbourne 2012 New York: Springer 305 311

[B2] Kim I Hur T Integration of working environment into life cycle assessment framework Int J Life Cycle Assess 2009 14 290 301 10.1007/s11367-009-0087-3

[B3] Pfeffer J Building sustainable organizations: the human factor Acad Manage Perspect 2010 24 34 45

[B4] Gray G Hartwell JK The chemical substitution tree Pollut Prvent Rev 1995 5 7 17

[B5] Ashford NA Industrial safety: the neglected issue in industrial ecology J Cleaner Prod 1997 5 115 121 10.1016/S0959-6526(97)00024-3

[B6] Armenti KR Moure-Eraso R Slatin C Geiser K Primary prevention for worker health and safety: cleaner production and toxics use reduction in Massachusetts J Cleaner Prod 2011 19 488 497 10.1016/j.jclepro.2010.07.006

[B7] National Research Council, Committee on Health Effects of Waste Incineration, Board on Environmental Studies and Toxicology, Commission on Life Sciences Waste Incineration & Public Health 2000 Washington, DC: National Academy Press

[B8] Hours M Anzivino-Viricil L Maitre A Perdix A Perrodin Y Charbotel B Bergeret A Morbidity among municipal solid waste workers: a cross-sectional study Int Arch Occ Env Hea 2003 76 467 472 10.1007/s00420-003-0430-0 12764617

[B9] U.S. Department of Health and Human Services, National Institute for Occupational Safety and Health Health Hazard Evaluation HETA 90-0329-2482 1995 New York: Centers for Disease Control and Prevention

[B10] Rosenberg BJ Barbeau EM Moure-Eraso R Levenstein C The work environment impact assessment: a methodologic framework for evaluating health-based interventions Am J Ind Med 2001 39 218 226 10.1002/1097-0274(200102)39:2<218::AID-AJIM1009>3.0.CO;2-4 11170164

[B11] Hofstetter NP Norris GA Why and how should we assess occupational health impacts in integrated product policy Environ Sci Technol 2003 37 2025 2035 10.1021/es025838w 12785504

[B12] Schmidt A Poulsen PB Andreasen J Fløe T Poulsen KE The working environment in LCA. A new approach Guidelines from the Danish Environmental Protection Agency No. 72 2004 Denmark: The Danish Environmental Protection Agency

[B13] Pettersen J Hertwich EG Occupational health impacts: offshore crane lifts in life cycle assessment Int J Life Cycle Assess 2008 13 440 449 10.1007/s11367-008-0003-2

[B14] International Organization for Standardization ISO 14040: Environmental Management, Life Cycle Assessment, Principles and Framework 2006 Switzerland: International Organization for Standardization

[B15] International Organization for Standardization ISO 14044: Environmental Management, Life Cycle Assessment, Requirements and Guidelines 2006 Switzerland: International Organization for Standardization

[B16] Murray CJL Lopez AD The Global Burden of Disease: A Comprehensive Assessment of Mortality and Disability from Diseases, Injuries, and Risk Factors in 1990 and Projected to 2020 1996 Boston: Harvard University Press

[B17] Hofstetter P Perspectives in Life Cycle Impact Assessment: A Structured Approach to Combine Models of the Technosphere, Ecosphere and Valuesphere 1998 Norwell: Kluwer Academic Publishers

[B18] U.S. Department of Labor, Bureau of Labor Statistics Occupational safety and health statistics BLS Handbook of Methodshttp://www.bls.gov/opub/hom/pdf/homch9.pdf

[B19] U.S. Department of Labor, Bureau of Labor Statistics National Census of Fatal Occupational Injuries in 2005, USDL 06–1364 2006 Washington, DC: News Release

[B20] U.S. Department of Labor, Bureau of Labor Statistics National Census of Fatal Occupational Injuries in 2006, USDL 07–1202 2007 Washington, DC: News Release

[B21] U.S. Department of Labor, Bureau of Labor Statistics National Census of Fatal Occupational Injuries in 2007, USDL 08–1182 2008 Washington, DC: News Release

[B22] U.S. Department of Labor, Bureau of Labor Statistics National Census of Fatal Occupational Injuries in 2008, USDL 09–0979 2009 Washington, DC: News Release

[B23] U.S. Department of Labor, Bureau of Labor Statistics National Census of Fatal Occupational Injuries in 2009 (Preliminary Results), USDL 10–1142 2010 Washington, DC: News Release

[B24] Arias E United States Life Tables, 2006 National Vital Statistics Reports **Volume 58**, Number 21 2010 Hyattsville, MD: U.S. Department of Health and Human Services 21043319

[B25] U.S. Department of Labor, Bureau of Labor Statistics Occupational Injury and Illness Classification Manual 2007 Washington, DC: U.S. Department of Labor

[B26] U.S. Department of Labor, Bureau of Labor Statistics Table 1b. Number and percent of nonfatal occupational injuries and illnesses involving days away from work by selected worker and case characteristics and gender, All United States, private industry, 2006 2007 Washington, DC: U.S. Department of Labor

[B27] Lopez AD Mathers CD Ezzati M Jamison DT Murray CJL Global Burden of Disease and Risk Factors 2006 New York: Oxford University Press 21250374

[B28] U.S. Department of Labor, Bureau of Labor Statistics, Injuries, Illnesses, and Fatalities Occupational Safety and Health Definitions: http://www.bls.gov/iif/oshdef.htm

[B29] Azaroff LS Levenstein C Wegman DH Occupational injury and illness surveillance: conceptual filters explain underreporting Am J Public Health 2002 92 1421 1429 10.2105/AJPH.92.9.1421 12197968 PMC1447253

[B30] Rosenman KD Kalush A Reilly MJ Gardiner JC Reeves M Luo Z How much work-related injury and illness is missed by the current national surveillance system? J Occup Environ Med 2006 48 357 365 10.1097/01.jom.0000205864.81970.63 16607189

[B31] Boden LI Ozonoff A Capture-recapture estimates of nonfatal workplace injuries and illnesses Ann Epidemiol 2008 6 500 506 18083542 10.1016/j.annepidem.2007.11.003

[B32] Ruser JW Examining evidence of whether BLS undercounts workplace injuries and illnesses Mon Labor Rev 2008 131 20 32

[B33] U.S. Government Accountability Office Workplace Safety and Health: Enhancing OSHA’s Records Audit Process could Improve the Accuracy of Worker Injury and Illness Data, GAO-10-10 2009 Washington, DC: U.S. Government Accountability Office

[B34] Nestoriak N Pierce B Comparing workers’ compensation claims with establishments’ responses to the SOII Mon Labor Rev 2009 132 57 64

[B35] Leigh JP Marcin JP Miller TR An estimate of the U.S. government’s undercount of nonfatal occupational injuries J Occup Environ Med 2004 46 10 18 10.1097/01.jom.0000105909.66435.53 14724473

